# MiR-20b Down-Regulates Intestinal Ferroportin Expression In Vitro and In Vivo

**DOI:** 10.3390/cells8101135

**Published:** 2019-09-24

**Authors:** Shuxia Jiang, Xi Fang, Mingni Liu, Yingdong Ni, Wenqiang Ma, Ruqian Zhao

**Affiliations:** 1 Key Laboratory of Animal Physiology and Biochemistry, Ministry of Agriculture and Rural Affairs, College of Veterinary Medicine, Nanjing Agricultural University, Nanjing 210095, Jiangsu, China; 2017207003@njau.edu.cn (S.J.); 2015107017@njau.edu.cn (X.F.); 2017107011@njau.edu.cn (M.L.); niyingdong@njau.edu.cn (Y.N.); zhaoruqian@njau.edu.cn (R.Z.); 2 MOE Joint International Research Laboratory of Animal Health & Food Safety, Nanjing Agricultural University, Nanjing 210095, Jiangsu, China; 3 National Center for International Research on Animal Gut Nutrition, Nanjing Agricultural University, Nanjing 210095, Jiangsu, China

**Keywords:** miR-20b, FPN, post-transcriptional regulation

## Abstract

Ferroportin (FPN) is the only known cellular iron exporter in mammalian. However, post-transcriptional regulation of intestinal FPN has not yet been completely understood. In this study, bioinformatics algorithms (TargetScan, PicTar, PITA, and miRanda) were applied to predict, screen and obtain microRNA-17 family members (miR-17, miR-20a, miR-20b, and miR-106a) targeting FPN, ‘seed sequence’ and responding binding sites on the 3′untranslated region (3′UTR) region of FPN. Dual-luciferase reporter assays revealed miRNA-17 family members’ mimics decreased the luciferase activity, whereas their inhibitors increased the luciferase activity. Compared with the FPN 3′UTR wild type reporter, co-transfection of a miRNA-17 family members’ over-expression plasmids and FPN 3′UTR mutant reporters enhanced the luciferase activity in HCT116 cells. Transfection with miR-20b overexpression plasmid significantly enhanced its expression, and it inhibited endogenous FPN protein expression in Caco-2 cells. Additionally, tail-vein injection of miR-20b resulted in increasing duodenal miR-20b expression, decreasing duodenal FPN protein expression, which was closely related to lower plasma iron level in mice. Taken together, these data suggest that the miR-20b is identified to regulate intestinal FPN expression in vitro and in vivo, which will provide a potential target for intestinal iron exportation.

## 1. Introduction

Ferroportin (FPN) is the only known cellular iron exporter in mammals and plays a role in maintaining iron homeostasis in cells and organism [1,2,3]. It is highly expressed in enterocytes, hepatocytes, macrophages, erythroid cells and placental syncytiotrophoblasts [1,3,4,5,6]. Loss- or gain-of-function of FPN disturbs iron export activity and induces iron metabolism disorders [7,8,9]. Loss-of-function of FPN resulted in restricted circulating iron and iron retention in the liver, spleen, and bone marrow macrophages, characterized by a compromised iron export from tissue macrophages [7,8,10,11]. Interfering with the FPN promoter region in mice has been reported to cause iron-deficiency anemia and transitory polycythemia [12]. Gain-of-function mutations of FPN (Q248H) impaired hepcidin-induced degradation of FPN, thereby leading to unrestricted circulating iron transfer and inducing a rare form of hereditary hemochromatosis in humans [9]. Besides, it was originally proposed that the FPN mutation of erythroid cells would be responsible for intracellular iron overload, cellular damage, and hemolysis in malaria-infected mice [9]. Controlling the regulation of FPN is extremely important for animal and human health.

The expression of FPN at the transcriptional level is regulated by hypoxia, heme, metal, and inflammation, post-transcriptionally by iron regulatory protein (IRP), and also translationally by hepcidin. Hypoxia-inducible factor-2 alpha (HIF2α) could bind to the HIF-responsive elements (HRE) located in the *Fpn* promoter region and acts as a direct activator of *Fpn* transcription [13]. Heme activates FPN transcription in an iron-independent manner through the transcriptional repressor btb and cnc homology 1(BACH1) and enhancer nuclear factor erythroid 2-like (NRF2), both of which containing a MAf recognition elements (MARE) antioxidant responsive elements (ARE) sequence motif located at position -7007/-7016 of the *Fpn* promoter [14,15]. Pro-inflammatory cytokine-mediated down-regulation of ferroportin transcription in liver, primary macrophages and neuronal cells under lipopolysaccharide (LPS)-induced acute inflammation [16,17]. IRPs bind to the iron regulatory elements (IRE) located in the 5′untranslated region (UTR) of FPN to decrease its mRNA stability, resulting in down-regulated expression of FPN protein, decrease export of iron, and enhance cellular iron accumulation [18,19,20]. Hepcidin, a systemic iron regulatory hormone, binds FPN for hepcidin-mediated internalization, ubiquitination, and subsequent degradation in the lysosome [5,21,22]. Additionally, miRNAs are considered to be important regulators that inhibit gene expression at the post-transcriptional level.

MiRNAs are a class of small non-coding RNAs that bind to the 3′untranslated region (3’ UTR) of target messenger RNA (mRNA) through complete or incomplete base pairing to negatively regulate gene expression [23]. Binding of miRNA to its target mRNA causes translation repression [24] and/or mRNA degradation [25]. Recent studies have shown that multiple miRNAs directly target specific genes involved in maintaining iron homeostasis [26]. MiR-320 and miR-152 target iron uptake related gene transferrin receptor 1 (TFR1) and inhibit cell proliferation in lung adenocarcinoma A549 cells and hepatocellular carcinoma, respectively [27,28]. MiR-let-7d induces iron accumulation in endosomes by suppressing expression of an isoform of divalent metal transporter 1 (DMT1) of K562 cells [29]. In vitro and in vivo studies showed that the miRNA-16 family (miR-15b, miR-16, miR-195 and miR-497) inhibits the expression of intestinal DMT1 [30]. MiR-485-3p and miR-20a regulate intracellular iron homeostasis by directly targeting FPN, an iron exporting gene in lung cancer and HepG2 cells, respectively [31,32]. However, the miRNAs targeting intestinal FPN remain elusive.

In this study, bioinformatics tools were used to predict and screen miRNAs targeting FPN, and the function of these candidate miRNAs was verified in vitro and in vivo.

## 2. Materials and Methods

### 2.1. Bioinformatic Analyses

MiRNA targeting FPN 3′UTR was predicted by common biological software: TargetScan (http://www.targetscan.org/), PicTar (http://pictar.mdc-berlin.de/), PITA (http://genie.weizmann.ac.il/pubs/mir07/mir07_data.html), and miRanda (http://www.microrna.org/microrna/home.do) and listed in Supplementary Table S1. For TargetScan, predictions were ranked according to the predictive efficacy of the target, using context++ scores of the binding sites [33]. In addition, predictions are also ranked according to the probability of their conservative targets [34]. PicTar predicted miRNA was based on the PicTar score and free energy [35]. PITA was implemented using high-stringiness parameters of 7-8 mers seeds, and setting energy value ≤ −10 kcal/mol as a general cutoff value [36,37]. miRanda was based on the following parameters: energy value ≤ −14 kcal/mol, score ≥ 80 [38].

### 2.2. Vector Construction

miRNAs overexpression plasmid (pcDNA3.1(+)-miRNAs), pmirGLO-FPN 3′UTR (the luciferase reporter vector constructs containing 1441 bp fragment of the FPN 3′UTR, XM_003483701.4) or pmirGLO-FPN 3′UTR mutants (mutated binding site of miRNAs on FPN 3′UTR) were synthesized by Zoonbio Biotechnology Co., Ltd. (Nanjing, Jiangsu, China). MiRNA inhibitors, as single-stranded 2′-O-methyl-modified RNA oligonucleotides, were synthesized by Biomics Biotechnologies Co., Ltd. (Nantong, Jiangsu, China). The miRNA inhibitors’ sequences are listed in Supplementary Table S2. All plasmids and miRNA inhibitors were used in the luciferase activity assay to detect the direct binding of miR-17, miR-20a, miR-20b, and miR-106a to target gene FPN.

### 2.3. Cell Culture

The human colon cancer cells (HCT116 and Caco-2) were purchased from Beijing Beina Chuanglian Biotechnology Institute (Beijing, China). HCT116 cells were cultured in RPMI-1640 (SH30809.01B, Hyclone, Logan, UT, USA). Caco-2 cells were cultured in DMEM (SH30243.01, Hyclone, Logan, UT, USA). Both cell lines were cultured in a medium containing 10% (*v*/*v*) FBS (A31608-02, Gibco, Carlsbad, CA, USA) and antibiotics (100 IU/mL penicillin and 100 IU/mL streptomycin) at 37 °C with 5% CO_2_. 1 × 10^5^/well HCT116 cells were inoculated into 24-well plates for luciferase reporter assay. 1 × 10^6^/well Caco-2 cells were inoculated into 6-well plates, and then 6 μg miRNA overexpressed plasmid was transfected into Caco-2 cells which were reached to 90~95% confluence 24 h later. After 24 h or 48 h transfection, cells were collected for Real-time PCR and Western blot analysis.

### 2.4. Luciferase Reporter Assay

When the HCT116 cells reached 90~95% confluence, 50 ng double luciferase reporter plasmid and 1 µg miRNA overexpression plasmids or scrambled miRNA plasmids (miR-SC, negative control of miRNA overexpression) were co-transfected with transfection reagent Lipofectamine 2000 (11668019, Life Technologies Inc., Waltham, MA, USA) for miRNA gain of-function assay. 50 ng double luciferase reporter plasmid, 1 µg miRNA overexpression plasmids and 1 µg miRNA inhibitors or miRNA inhibitors NC (negative control of miRNA inhibitors) were co-transfected with transfection reagent Lipofectamine 2000 for miRNA loss of-function assay. Twenty-four hours after transfection, luciferase activity was detected on the GlOMAXTM 96 microplate luminometer (Promega, Madison, WI, USA) using the dual-luciferase reporter assay system (E1910, Promega, Madison, WI, USA), according to the manufacturer’s instructions. The same method was employed for FPN 3′UTR mutated forms.

### 2.5. Experimental Animals

A total of 30 male SPF C57BL/6J mice (16~18 g) were purchased from Shanghai Ling Chang Biological Technology Co., Ltd. at six weeks of age. The animals were housed at a constant temperature of 22 ± 1 °C with a 12 h light/dark cycle, and given free access to both food and deionized water. After 1 week of acclimatization, mice were divided into a negative control group (miR-SC, *n* = 15) and a miR-20b over-expression group (miR-20b, *n* = 15) and were subjected to the same diets. Plasmid transfection was performed with some modifications based on previous studies [30]. Briefly, 20 μg pcDNA3.1(+)-miRNAs plasmid (miR-SC or miR-20b) in 150 μL Opti-MEM medium (31985-070, Gibco, Carlsbad, California, USA) was mixed with 25 μL lipofectamine 2000 in 150 μL Opti-MEM medium, and the total mixture (300 μL) was incubated for 30 min at room temperature. Meanwhile, mice were injected with plasmids via the tail-vein once every two days for a total of five times. Body weight and feed intake were recorded throughout the feeding period to calculate average daily feed intake. At the endpoint, mice were anesthetized with an intraperitoneal injection of 1% pentobarbital sodium after overnight fasting. Blood samples were collected from the abdominal aorta and stored at −20 °C for determination of iron content in plasma. Duodenum samples were rapidly harvested and frozen at −80 °C. All the procedures were conducted in accordance with the guidelines of the Animal Ethics Committee of Nanjing Agricultural University.

### 2.6. Determination of Iron Concentration

Plasma iron content was detected by the automatic biochemical analyzer (7020, Hitachi High-Tech Corporation, Tokyo, Japan) following the instructions of the kit (6063-2012, Shino-Test Corporation, Tokyo, Japan). The content of iron in the diet was determined by atomic absorption spectrometry based on the method described previously [39].

### 2.7. RNA Isolation and Quantification of Mature MiRNAs

Initially, total RNA was extracted from the cultured cells with the TRIzol reagent (15596026, Invitrogen, Carlsbad, CA, USA) following the manufacturer’s instruction. Total RNA concentrations were measured by NanoDrop-1000 spectrophotometer and treated with RNase-Free DNase I (D2215, Takara, Otsu, Japan). MiRNA analysis was performed using our previous publication with some modifications [40,41]. 6 μg of total RNA were polyadenylated with poly (A) polymerase for 1 h at 37 °C in a 25 μL reaction mixture using Poly (A) Tailing Kit (AM1350, Applied Biosystems, Waltham, MA, USA) according to the manufacturer’s instructions. 2 μg of polyadenylated RNA were reverse transcribed using the poly (T) adapter. Real-time quantitative PCR was performed in an Mx3000P real-time PCR detection system (Stratagene, La Jolla, CA, USA) with AceQ qPCR SYBR Green Master Mix (Q111-02, Vazyme, Nanjing, Jiangsu, China) using a miRNA-specific forward primer and a universal reverse primer. Exogenous reference was used as a reference gene to normalize the expression of miRNAs. The sequences of all the primers, poly (T) adapters, exogenous reference genes are listed in Supplementary Table S3.

### 2.8. Quantitative Real-Time PCR

After RNA extraction and quality verification, the RNA concentration was unified to 500 ng/μL per sample, then it was reversely transcribed into cDNA with HiScript II Q RT SuperMix (R223-01, Vazyme, Nanjing, Jiangsu, China) following the manufacturer’s instructions. A total of 2 µL of diluted cDNA (1:25, *v*/*v*) was used as a template in PCR reactions on a real-time PCR system (Mx3000P, Stratagene, La Jolla, CA, USA). Primers specific for FPN (GenBank No. NM_016917.2) were used for the quantification (F: 5′-AGTCATTGGCTGTGGTTT-3′, R: 5′-TTTGGCTCAGTATCTTTAGGT-3′), whereas primers specific for the peptidylprolyl isomerase A (Ppia) (GenBank No. NM_008907.1) were selected as a reference gene in duodenum (F: 5′-GGGTTCCTCCTTTCACAGA-3′, R: 5′-CCATCCAGCCATTCAGTC-3′). Relative gene expression was calculated with 2^-ΔΔCt^ method.

### 2.9. Protein Extraction and Western Blot Analysis

Total protein from cells or tissue samples was extracted using 1×RIPA buffer [42] containing protease inhibitor (P8340, Sigma, St. Louis, MO, USA). Then the lysate was incubated for 30 min on ice and centrifuged at 12,000 rpm for 15 min at 4 °C. The protein concentration was measured using Pierce BCA Protein Assay Kit (23225, Thermo Scientific, Waltham, PA, USA). Then, 70 µg protein from each sample was separated using SDS-polyacrylamide gel electrophoresis (10%, *w*/*v*) and blotted onto nitrocellulose membrane. The membranes were blocked in 5% (*v*/*v*) skimmed milk for 2 h, and then incubated overnight at 4 °C with diluted primary antibodies: FPN (sc-49668, Santa Cruz, 1:200), β-actin (AP0060, Bioworld, 1:10,000). After three times washes (10 min each time) in TBST with 0.1% Tween at room temperature, secondary antibodies were incubated for 2 h at room temperature. Following extensive washing with TBST, the ECL signal intensities were quantified using the VERSADOC MP 4000 system (Bio-Rad, California, CA, USA). Antibody against β-actin served as an endogenous reference.

### 2.10. Statistical Analysis

The graphical presentations were prepared using GraphPad Prism 5.0. Data were analyzed using One-Way ANOVA in SPSS Statistics (Chicago, IL, USA) and presented as the mean ± SEM. Differences were considered significant at *p* < 0.05.

## 3. Results 

### 3.1. Computational Tools Screen and Predict MiRNAs Targeting FPN

**Figure 1:**
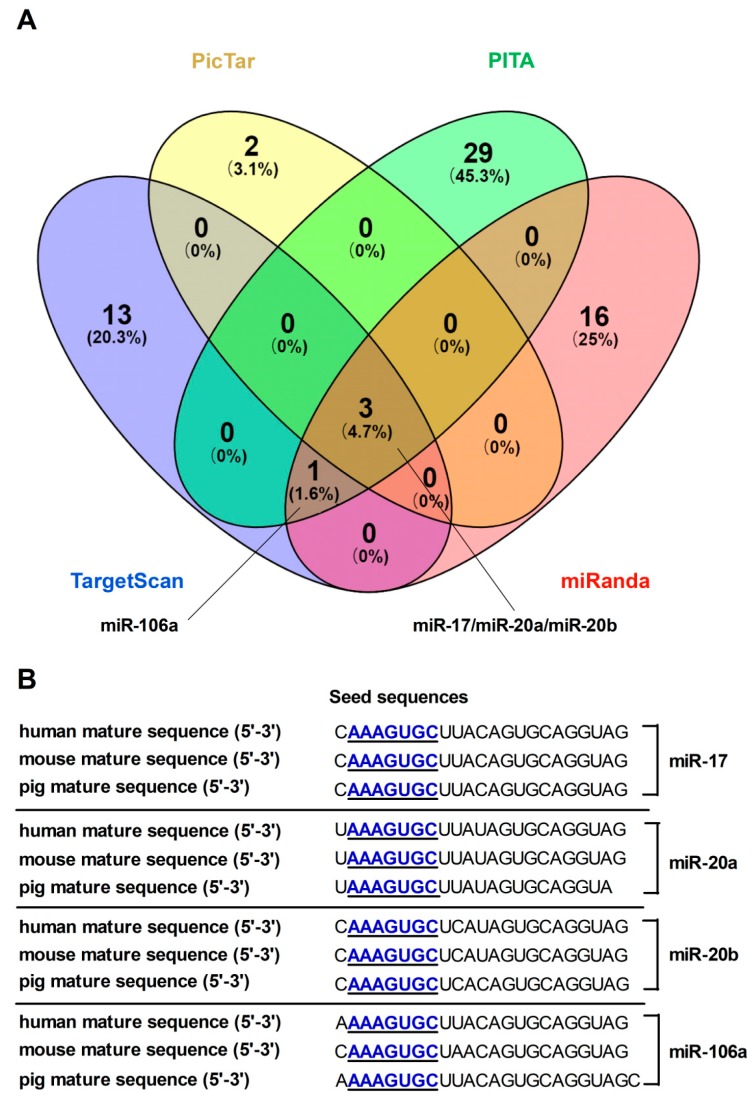
Computational prediction of potential miRNAs target sites on the ferroportin (FPN) 3’untranslated region (3’UTR). (**A**) miRNAs predicted from four different bioinformatic tools were compared using a Venn diagram (**B**) The seed recognition sites of candidate miRNAs are denoted, and all nucleotides in these regions are highly conserved across species.

To find out the potential miRNAs, bioinformatics programs (TargetScan, PicTar, PITA, and miRanda) were applied to screen and predict miRNAs targeting FPN. MiR-17, miR-20a, miR-20b, and miR-106a were identified as FPN targeting miRNAs through the intersection of every three bioinformatics programs (Figure 1A). To facilitate functional identification, we analyzed ‘seed sequences’ of these miRNAs and its binding sites within the FPN 3′UTR, which were highly conserved among mammals (Figure 1B).

### 3.2. FPN mRNA Is the Target of Candidate MiRNAs

**Figure 2:**
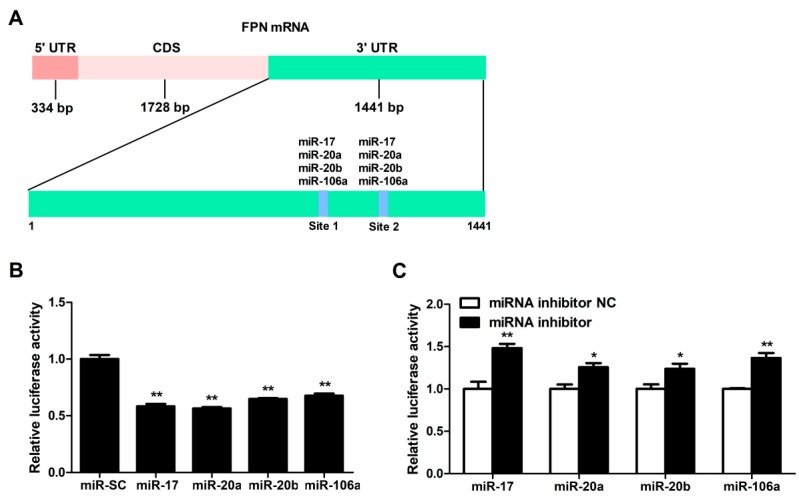
FPN mRNA is the target of candidate miRNAs. (**A**) Prediction of binding sites for miR-17, miR-20a, miR-20b, and miR-106a on the FPN 3′UTR by bioinformatics algorithms. (**B**) FPN 3′ UTR Luciferase reporter activity was measured 24 h after transfections of miR-17, miR-20a, miR-20b, miR-106a and miR-NC in HCT116 cells. (**C**) Fold change in luminescence of FPN 3′ UTR luciferase reporter of miR-17, miR-20a, miR-20b, and miR-106a inhibition relative to control (miRNA inhibitor NC) for 24 h. Values were expressed as means ± SEM, *n* = 4 in each group, * *p* < 0.05, ** *p* < 0.01.

These candidate miRNAs were analyzed to bind two identical binding sites on the 3′UTR sequence of FPN (Figure 2A). To investigate whether FPN 3′UTR is targeted by these miRNAs, we co-transfected these miRNAs overexpression plasmids and FPN 3′UTR luciferase reporter gene into cells, then detected the luciferase activity of FPN 3′UTR reporter with luciferase activity assay. The results indicated that delivery of the miRNA-17 family members (miR-17, miR-20a, miR-20b, and miR-106a) mimics led to a decrease of the FPN 3′UTR luciferase reporter activity (*p* < 0.01, Figure 2B), while delivery of either of these four inhibitors led to an increase in FPN 3′UTR luciferase reporter activity (*p* < 0.05 or *p* < 0.01, Figure 2C).

### 3.3. MiRNA-17 Family Members (miR-17, miR-20a, miR-20b, and miR-106a) Directly Bind the 3′UTR of FPN

**Figure 3:**
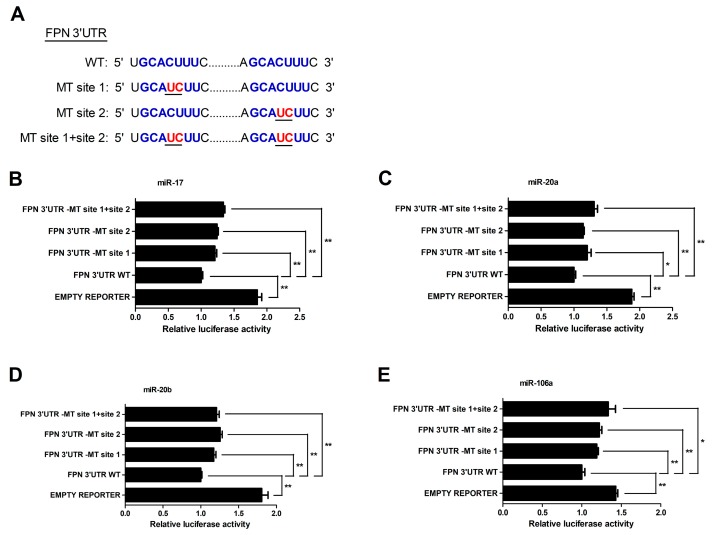
MiR-17, miR-20a, miR-20b, and miR-106a directly bind the 3′UTR of FPN in HCT116 cells. (**A**) Schematic illustration of FPN 3′ UTR WT and FPN 3′ UTR-MT sequences were cloned downstream of a luciferase reporter. (**B**–**E**) The relative luciferase activity of FPN 3′ UTR-MT site 1, FPN 3′ UTR-MT site 2, FPN 3′ UTR-MT site 1+site 2 and empty luciferase reporter compared to FPN 3′ UTR WT. Values were expressed as means ± SEM, *n* = 4 in each group, * *p* < 0.05, ** *p* < 0.01.

To further determinate whether the FPN 3′UTR is a direct target of miRNA-17 family members (miR-17, miR-20a, miR-20b and miR-106a), we created mutant FPN 3′UTR reporters with mutations in the binding sites (MT site 1, MT site 2, or MT site 1 + site 2) (Figure 3A). The results showed that the luciferase activity of the mutant FPN 3′UTR reporter was lower than that of the empty vector. However, compared with the wild type FPN 3′UTR reporter, mutation of binding sites resulted in an increase of the luciferase reporter activity (*p* < 0.05 or *p* < 0.01, Figure 3B–E), indicating that the two binding sites served as an important role in miRNA-17 family-induced regulation of the FPN.

### 3.4. Candidate MiRNAs Overexpressed in Caco-2 Cells

**Figure 4:**
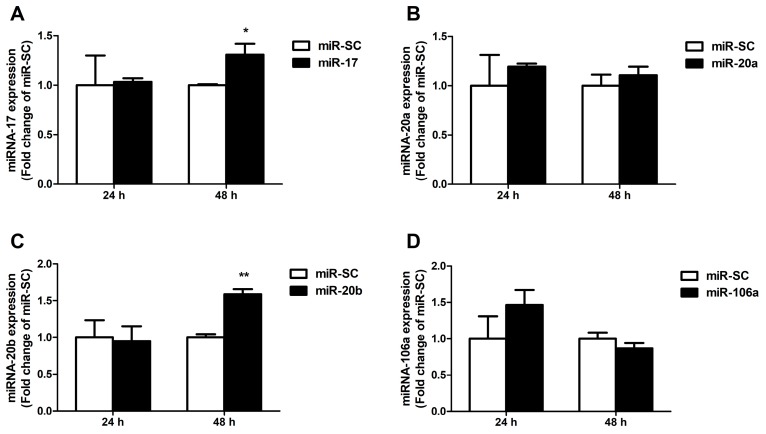
Expression of the selected miRNAs in Caco-2 cells. (**A**) The results of a quantitative RT-PCR analysis of the miR-17 expression level in Caco-2 cells transfected for 24 h and 48 h. (**B–D**) The levels of the miR-20a, miR-20b and miR-106a in Caco-2 cells were measured by qRT-PCR at 24 h and 48 h after transfection. Values were expressed as means ± SEM, *n* = 3 in each group, * *p* < 0.05, ** *p* < 0.01.

Next, miRNA-17 family members were overexpressed in cells by transient transfection of miR-17 family overexpression plasmids for 24 h and 48 h. Results indicate that overexpression of miR-17 and miR-20b strongly elevated cellular miR-17 and miR-20b expression at 48 h, but did not affect both of their expression at 24 h (*p* < 0.01, Figure 4A,C). Overexpression of miR-20a and miR-106a did not affect miR-20a and miR-106a expression at 24 h and 48 h in Caco-2 cells (Figure 4B,D).

### 3.5. Up-Regulation of MiR-20b Suppressed Endogenous FPN Expression

**Figure 5:**
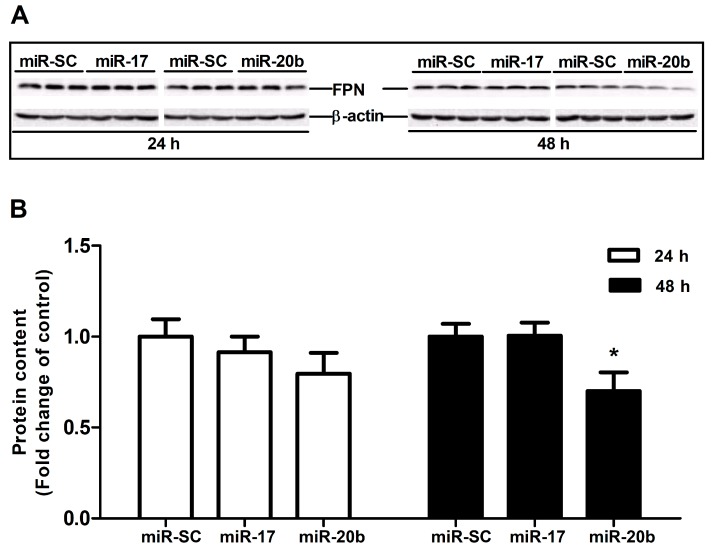
Effect of miR-17 and miR-20b on endogenous FPN expression in vitro. (**A**) The protein expression of FPN was evaluated by Western blot in Caco-2 cells transfected with miR-17, miR-20b, and vector control (miR-SC) for 24 h and 48 h. (**B**) Bar graphs representing quantification of FPN protein expressions normalized to β-actin. Values were expressed as means ± SEM, *n* = 6 in each group, * *p* < 0.05.

To confirm miRNA-target interactions, miR-17 and miR-20b over-expression plasmid was transfected into Caco-2 cells to evaluate FPN protein expression at 24 h and 48 h. The results showed that overexpression of miR-17 had no influence on FPN protein expression at both 24 h and 48 h. miR-20b overexpression did not affect FPN protein expression at 24 h, whereas it markedly reduced the protein expression of FPN at 48 h (*p* < 0.05, Figure 5A,B).

### 3.6. MiR-20b Down-Regulates Intestinal FPN Expression In Vivo

**Figure 6:**
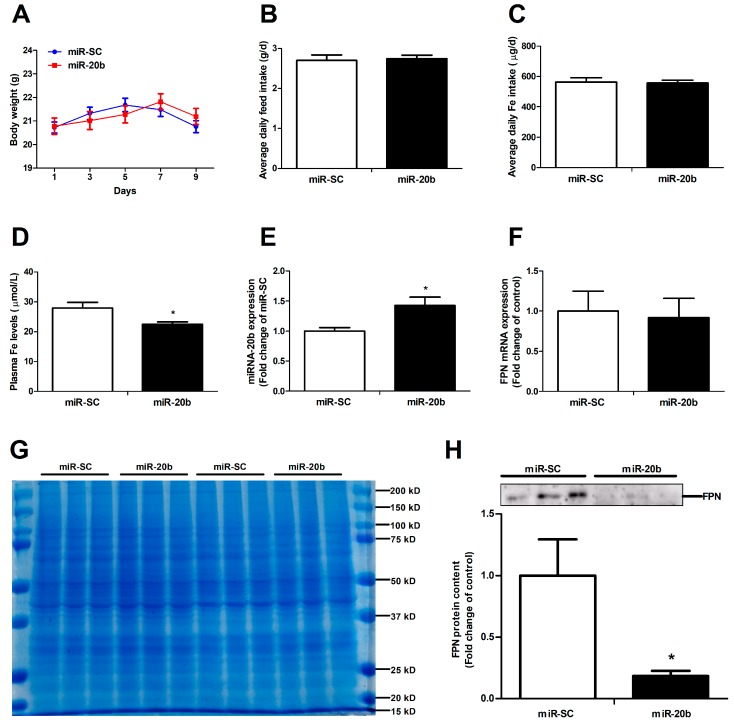
MiR-20b regulates endogenous FPN expression in vivo. (**A**) Body weight was calculated every two days. (**B**,**C**) Average daily feed intake and average daily iron intake throughout the feeding period. (**D**) Plasma iron level was measured by an automatic biochemical analyzer. (**E**) Expression of miR-20b in the duodenum of mice was analysed by qRT-PCR. (**F**) The mRNA expression of FPN was evaluated by qRT-PCR. (**G**) Coomassie brilliant blue (CBB) staining ensures that equal amounts of duodenal protein samples are loaded onto the gel. (**H**) FPN protein expression was analyzed by western blot. Values were expressed as means ± SEM, *n* = 15 in each group for Figure 6A–D, *n* = 6 in each group for Figure 6E–H, * *p* < 0.05.

Functional miR-20b was validated in vivo via the tail-vein injection in mice. It turned out that miR-20b overexpression did not affect the body weight (Figure 6A), average daily feed intake (Figure 6B), and average daily iron intake (Figure 6C) in mice. MiR-20b plasmid injection dramatically decreased the plasma iron level and increased the duodenal miR-20b expression in mice (*p* < 0.05, Figure 6D,E). Up-regulation of miR-20b had no effect on duodenal FPN mRNA expression, but significantly reduced duodenal FPN protein expression in mice (*p* < 0.05, Figure 6F,H). Coomassie brilliant blue (CBB) is a dye used for the visualization of equal amounts of duodenal protein samples (Figure 6G). In addition, miR-20b did not impact on hepatic FPN protein expression in mice (Data not shown). 

## 4. Discussion

Computational tools are useful for analyzing and identifying predicted interactions between regulatory miRNAs and their target genes. TargetScan, PicTar, PITA and miRanda proved to be successful bioinformatic algorithms for miRNA prediction [34,36,43,44]. Four miRNAs (miR-17, miR-20a, miR-20b and miR-106a) targeting FPN were screened and selected basing on crosstalk among these prediction algorithms, which belongs to miRNA-17 family according to the miRNA ‘seed sequence’ [45]. Moreover, the mature sequence and its ‘seed sequence’ of these four miRNAs were highly conserved among human, mouse, and pig based on identification of bioinformatics databases: miRbase [46], TargetScan [34], PicTar [43], PITA [36] and miRanda [44]. It is reported that miRNA-17 family plays a critical role in normal cardiac development, cardiovascular disease and polycystic kidney disease progression as well as affecting hepatic steatosis and insulin signaling [47,48,49,50,51,52]. Babu et al. (2016) revealed that enhancing expression of miR-20a could decrease iron export and increase intracellular iron retention in lung cancer [32]. Nevertheless, the regulation of four miRNAs targeting intestinal FPN needs further verification in multiple ways based on tissue-specific miRNA expression pattern. 

Iron is absorbed and transported into the systemic circulation through the proximal small intestinal epithelium, primarily the duodenum in mammals [53,54]. In recent years, no mature duodenal cell lines have been found, while HCT116 and Caco-2 cells are commonly used for intestinal iron absorption and transportation [55,56,57,58,59]. Moreover, there are many studies on the function of miRNAs in HCT116 and Caco-2 cells. MiR-31 as a critical modulator of intestinal stem cells (ISC) biology, and a potential therapeutic target for a broad range of intestinal regenerative disorders and cancers [60]. MiR-29a may regulate the radiosensitivity of intestinal cell lines by targeting the phosphatase and tensin homologue deleted on chromosome-10 (PTEN) gene [61]. MiR-106b targets autophagy related gene 16L1 (ATG16L1) and modulates autophagy in intestinal epithelial HCT116 cells [62]. Long Noncoding RNA uc.173 regulates growth of the intestinal mucosa and stimulates intestinal epithelial renewal by reducing level of miR-195 [63]. MiR-103a regulates sodium-dependent vitamin C transporter-1 expression in intestinal epithelial cells [64]. MiR-584 mediates post-transcriptional expression of lactoferrin receptor in Caco-2 cells and in mouse small intestine during the perinatal period [65]. In addition, the proportion of endogenous miRNA-17 family in HCT116 cells was lower than that in other cells [66,67]. Therefore, HCT116 and Caco-2 cells were used for the targeting and functional verification of miRNA-17 family targeted intestinal FPN in vitro, respectively.

MiRNAs are processed, and then formed to the effector RNA-induced silencing complex (RISC), which act by targeting specific mRNAs for degradation or translation repression [23,68,69]. Dual-luciferase 3′UTR reporter assays could be applied to identify miRNA-mRNA interaction, providing functional evidence for a miRNA’s effect on the 3′UTR and reflecting the changes in protein abundance of targeted genes [70,71,72,73]. Synthetic miRNA mimics (inhibitors) are induced into cells, which will enhance (reduce) endogenous miRNA activity representing a gain (loss)-of-function assay [74,75,76,77]. The synthetic mimics are loaded into RISC which enables regulation of mRNA primarily through partial complementarity to target sites in the 3′UTR of a particular transcript [78]. The inhibitors comprise a non-hydrolyzable, single-strand reverse complement to the mature miRNA strand to prevent binding of the mature miRNA to its endogenous targets [79]. Using the dual-luciferase reporter vector containing FPN 3′ UTR, delivery of miRNA-17 family members’ mimics resulted in a decrease of luciferase activity, while delivery its inhibitor resulted in enhancement of luciferase activity. MiRNA-mediated down-regulation of FPN did not show a linear relationship between the binding sites and the magnitude of repression, which was closely related to the binding affinity for each miRNA-target site interaction [67,80,81,82]. Further, the target gene is known not only to be regulated by specific individual miRNA but also multiple endogenous miRNAs [67,83]. Therefore, luciferase assays revealed that the miRNA-17 family member overexpression down-regulated activity of FPN-3′UTR-wild type reporter, but in a non-linear way, luciferase activity inhibition was reversed by FPN 3′UTR mutant reporters with point mutations of miRNA-17 family target site. Such gain- and loss-of-function data strongly indicates that the FPN 3′UTR is directly regulated by the miRNA-17 family members.

The expression of miR-17 and miR-20b was significantly increased after miR-17 or miR-20b overexpression for 48 h, but only miR-20b were likely to interact with the 3′ UTR of endogenous FPN, and consequently down-regulated its expression at the posttranscriptional level. It is reported that the transfected exogenous miRNAs and endogenous miRNAs competed for available RISC binding, which resulted in dysregulation of target genes, even upregulation of corresponding mRNAs and proteins [67,83,84]. In addition, an individual miRNA could post-transcriptionally regulate the expression of multiple genes [85]. Therefore, competing with endogenous miRNAs and multiple targets may play a role in disturbed FPN expression after miR-17 transfection for 48 h. 

Intravenous tail-vein injection of overexpressed genes displays a way of measuring function to targeting genes in vivo [86,87]. In vivo miR-155 inhibition induced by anti-miR-155 injections resulted in an increase in expression of genes that bind to it in the brain, including SMAD5, Rictor, and eNOS [86]. Tail-vein injection of miR-200a/200b mimics decreased endothelial O-GlcNAc transferase (OGT) and intercellular adhesion molecule 1 (ICAM-1) expression in db/db mice [87]. In this study, direct injection of the miR-20b overexpression plasmid resulted in a significant increase in miR-20b expression and a great reduction in duodenal FPN protein expression in mice. Decreasing plasma iron level is closely linked to reduced duodenal FPN protein expression, which will lower the export efficiency of ferrous iron from the intestinal lumen into the blood [2,3]. Unchanged hepatic FPN protein expression was probably induced by endogenous RNA. MiRNA repression can be inhibited by competing endogenous RNA (ceRNA), which perturbs miRNA function via sharing miRNA binding sites [88,89].

## 5. Conclusions

In summary, the present data indicate that the miR-20b is identified to regulate intestinal FPN expression in vitro and in vivo, which will provide a potential target for intestinal iron exportation.

## Supplementary Materials

The following are available online at https://www.mdpi.com/2073-4409/8/10/1135/s1. Table S1. miRNAs targeted FPN prediction using TargetScan, PicTar, PITA and miRanda, Table S2. miRNA inhibitors’ sequences, Table S3. miRNA and the corresponding primer sequences.
