# Supplementary material for: MiR-20b Down-Regulates Intestinal Ferroportin Expression In Vitro and In Vivo
Source: Cells. 2019 Sep 24;8(10):1135. doi: 10.3390/cells8101135 (PMC6829237; doi:10.3390/cells8101135)
Supplement: Supplementary file 1 [file cells-08-01135-s001.zip › Supplementary materials.doc]

**Supplementary materials**

Supplementary materials contain Supplementary Tables S1–S3.

**Table 1.** miRNAs targeted FPN prediction using TargetScan, PicTar, PITA and miRanda.

| **NO.** | **TargetScan** | **PicTar** | **PITA** | **miRanda** |
| --- | --- | --- | --- | --- |
| 1 | miR-221 | **miR-20a** | **miR-20a** | **miR-20a** |
| 2 | miR-222 | miR-106b | **miR-20b** | **miR-20b** |
| 3 | **miR-20a** | **miR-17** | miR-324-3p | **miR-106a** |
| 4 | miR-302e | miR-372 | miR-1225-5p | **miR-17** |
| 5 | **miR-20b** | **miR-20b** | miR-574-5p | miR-539 |
| 6 | **miR-106a** |  | miR-147 | miR-519d |
| 7 | miR-302a |  | miR-432 | miR-454 |
| 8 | **miR-17** |  | miR-330-3p | miR-301a |
| 9 | miR-93 |  | miR-575 | miR-301 |
| 10 | miR-302c |  | miR-1266 | miR-590-3p |
| 11 | miR-302b |  | miR-143 | miR-124 |
| 12 | miR-302d |  | miR-744 | miR-506 |
| 13 | miR-520b |  | miR-644 | miR-544 |
| 14 | miR-520c-3p |  | miR-26a | miR-300 |
| 15 | miR-520d-3p |  | miR-26b | miR-381 |
| 16 | miR-520a-3p |  | miR-1272 | miR-132 |
| 17 | miR-520e |  | **miR-106a** | miR-212 |
| 18 |  |  | **miR-17** | miR-144 |
| 19 |  |  | miR-1294 | miR-376c |
| 20 |  |  | miR-936 | miR-101 |
| 21 |  |  | miR-1184 |  |
| 22 |  |  | miR-1207-5p |  |
| 23 |  |  | miR-502-5p |  |
| 24 |  |  | miR-1231 |  |
| 25 |  |  | miR-410 |  |
| 26 |  |  | miR-122 |  |
| 27 |  |  | miR-508-3p |  |
| 28 |  |  | miR-593 |  |
| 29 |  |  | miR-501-3p |  |
| 30 |  |  | miR-518a-5p |  |
| 31 |  |  | miR-527 |  |
| 32 |  |  | miR-449b |  |
| 33 |  |  | miR-512-3p |  |

Note: The microRNAs predicted to target FPN are shown using four prediction tools including TargetScan (Context++ score percentile ≥ 80), PicTar, PITA (ddG score ≤ -10) and miRanda (ddG score ≤ -14). In bold are overlapping microRNAs of the prediction results.

**Table 2.** miRNA inhibitors sequences.

| **Names** | **Sequence** | | **miRbase Accession** |
| --- | --- | --- | --- |
|  | sense（5'-3'） | antisense（5'-3'） |  |
| **miR-17 inhibitor** | caaagugcuuacagugcagguag | cuaccugcacuguaagcacuuug | MIMAT0000070 |
| miR-20a inhibitor | uaaagugcuuauagugcagguag | cuaccugcacuauaagcacuuua | MIMAT0000075 |
| miR-20b inhibitor | caaagugcucauagugcagguag | cuaccugcacuaugagcacuuug | MIMAT0001413 |
| miR-106a inhibitor | aaaagugcuuacagugcagguag | cuaccugcacuguaagcacuuuu | MIMAT0000103 |
| miRNA inhibitor NC | ucacaaccuccuagaaagaguaga | ucuacucuuucuaggagguuguga | N/A |

**Table 3. miRNA and the corresponding primer sequences.**

| **Names** | **Sequence (5’to 3’)** | **miRbase Accession** |
| --- | --- | --- |
| miR-17 | caaagtgcttacagtgcaggtag | MIMAT0000070 |
| miR-20a | taaagtgcttatagtgcaggtag | MIMAT0000075 |
| miR-20b | caaagtgctcacagtgcaggtag | MIMAT0001413 |
| miR-106a | aaaagtgcttacagtgcaggtag | MIMAT0000103 |
| Universal primer | tagagtgagtgtagcgagca | N/A |
| Poly(T) adapter | tagagtgagtgtagcgagcacagaattaatacgactcactatagg(t)16vn | N/A |
| Exogenous reference | gtgacccacgatgtgtattcgc | N/A |
